# Anthelminthic treatment during pregnancy is associated with increased risk of infantile eczema: randomised-controlled trial results

**DOI:** 10.1111/j.1399-3038.2010.01122.x

**Published:** 2011-05

**Authors:** Harriet Mpairwe, Emily L Webb, Lawrence Muhangi, Juliet Ndibazza, Denise Akishule, Margaret Nampijja, Sophy Ngom-wegi, Josephine Tumusime, Frances M Jones, Colin Fitzsimmons, David W Dunne, Moses Muwanga, Laura C Rodrigues, Alison M Elliott

**Affiliations:** 1 MRC/UVRI Uganda Research Unit on AIDSEntebbe, Uganda; 2 London School of Hygiene and Tropical MedicineLondon, UK; 3 Entebbe HospitalEntebbe, Uganda; 4 Department of Pathology, University of CambridgeCambridge, UK

**Keywords:** albendazole, praziquantel, worms, infantile eczema, pregnancy, clinical trial

## Abstract

**Background:**

Allergy is commoner in developed than in developing countries. Chronic worm infections show inverse associations with allergy, and prenatal exposures may be critical to allergy risk.

**Objective:**

To determine whether anthelminthic treatment during pregnancy increases the risk of allergy in infancy.

**Methods:**

A randomised, double-blind, placebo-controlled trial on treatment in pregnancy with albendazole versus placebo and praziquantel versus placebo was conducted in Uganda, with a 2 × 2 factorial design; 2507 women were enrolled; infants’ allergy events were recorded prospectively. The main outcome was doctor-diagnosed infantile eczema.

**Results:**

Worms were detected in 68% of women before treatment. Doctor-diagnosed infantile eczema incidence was 10.4/100 infant years. Maternal albendazole treatment was associated with a significantly increased risk of eczema [Cox HR (95% CI), p: 1.82 (1.26–2.64), 0.002]; this effect was slightly stronger among infants whose mothers had no albendazole-susceptible worms than among infants whose mothers had such worms, although this difference was not statistically significant. Praziquantel showed no effect overall but was associated with increased risk among infants of mothers with *Schistosoma mansoni* [2.65 (1.16–6.08), interaction p = 0.02]. In a sample of infants, skin prick test reactivity and allergen-specific IgE were both associated with doctor-diagnosed eczema, indicating atopic aetiology. Albendazole was also strongly associated with reported recurrent wheeze [1.58 (1.13–2.22), 0.008]; praziquantel showed no effect.

**Conclusions:**

The detrimental effects of treatment suggest that exposure to maternal worm infections *in utero* may protect against eczema and wheeze in infancy. The results for albendazole are also consistent with a direct drug effect. Further studies are required to investigate mechanisms of these effects, possible benefits of worms or worm products in primary prevention of allergy, and the possibility that routine deworming during pregnancy may promote allergic disease in the offspring.

The World Health Organisation (WHO) estimates that 20% of the world population now suffers from allergic diseases (1), but this epidemic is no longer restricted to developed countries: low and lower-middle income countries experience over 80% of asthma deaths worldwide (2).

Many factors may be involved in the global increase in allergic disease, but the declining incidence and prevalence of infectious diseases is widely considered to be an important contributor (3). Chronic worm infections may particularly protect against allergy (4). During established infections, worms evoke strong immunoregulation, protecting themselves against host responses, for example, by eliciting regulatory cytokines such as interleukin (IL)-10 and transforming growth factor (TGF-) β. ‘Bystander’ effects of these processes may down-regulate responses to other immunogens, including allergens (5). In humans, several cross-sectional studies have demonstrated inverse associations between chronic worm infections and atopy (6,7), and initial intervention studies suggested that regular anthelminthic treatment was associated with increased skin prick reactivity (8). However, a large trial among school children in Ecuador showed no such effect: if worm infections protect against allergy, their effect, at least on skin prick reactivity, could not readily be reversed at this age (9). One contributory explanation to this paradox could be that long-term, worm-mediated effects are established earlier in life, and indeed, there is considerable evidence that prenatal exposures are critical to later susceptibility to allergic disease (10).

Worm infections during pregnancy also have important immunological effects on the offspring, including sensitisation to worm antigens (11), long-term effects on the response to infection with the same worm species (12), and modification of responses to unrelated vaccine antigens (13). Whether worm infections during pregnancy influence the response to allergens or development of allergy in the offspring is not known. This question is particularly topical in view of current promotion of policies for deworming during pregnancy WHO recommended mass treatment for soil transmitted helminths during pregnancy (with drugs including albendazole) in areas where the prevalence of hookworm is more than 20% in 1994 (14) and the use of praziquantel for treatment of schistosomiasis in pregnancy in 2002 (15), with the subsequent provision that this should be investigated in placebo-controlled trials (16).

Our preliminary study showed a reduced risk of eczema among infants whose mothers had worms and suggested an increased incidence among infants of mothers who received albendazole during pregnancy compared to infants whose mothers received placebo (17). We now present results of a large randomised-controlled trial of treatment during pregnancy with albendazole and praziquantel, addressing the hypothesis that worm infections during pregnancy protect against allergic diseases in infancy and that treatment of worms during pregnancy increases their incidence.

## Methods

### Setting and participants

The study has been described [ISRCTN32849447] (18). It was conducted in Wakiso district, Uganda. Pregnant women attending Entebbe Hospital (a free Government hospital serving most of the local community) for their first antenatal visit between April 2003 and November 2005 were screened for enrolment. Women were eligible if they were well, in their second or third trimester, resident in the study area, planning delivery at Entebbe Hospital, willing to participate and willing to know their HIV status and excluded if they had haemoglobin <8 g/dl, clinically apparent severe liver disease, diarrhoea with blood in the stool, abnormal pregnancy, history of adverse reaction to anthelminthic drugs, or had participated in the study during an earlier pregnancy. All participants gave informed written consent.

The study was approved by ethics committees of the Uganda Virus Research Institute and London School of Hygiene and Tropical Medicine, and by the Uganda National Council for Science and Technology.

### Study design

This was a randomised, double-blind, placebo-controlled trial of antheminthics in pregnancy, using albendazole versus placebo and praziquantel versus placebo in a 2 × 2 factorial design. Prior to enrolment, participants provided a single stool and blood samples for parasitology and other investigations. At enrolment, they were allocated to one of the four treatment arms (albendazole + praziquantel, albendazole + praziquantel-placebo, albendazole-placebo + praziquantel, albendazole-placebo + praziquantel-placebo) according to a random sequence generated in blocks of 100 by the trial statistician. Albendazole tablets (or matching placebo) and 12 capsules of praziquantel (or matching placebo) were provided in sealed, identical, opaque envelopes, labelled according to the allocation sequence, and used sequentially to administer a single dose of albendazole (400 mg) (or placebo) and of praziquantel (40 mg/kg) (or placebo) under observation by study staff. Participants and staff were blinded to treatment allocation. Following delivery, all women provided another stool sample to assess the effect of therapy. At 6 wk post-delivery, all women received albendazole and praziquantel and additional treatment for specific worm species, such as *Strongyloides*, if required. Infants were seen at 6, 10 and 14 wk, 6, 9 and 12 months and at interim illness events.

### Outcomes

The study was originally designed to examine the effects of anthelminthic treatment during pregnancy on infant responses to immunisation and infectious disease incidence. However, data on all illness events, including doctor-diagnosed allergy, were collected from the start of the trial and reported allergy outcomes were added to the 1-yr visit questionnaire before any participating child had reached age 1 yr, in response to our initial findings (17), as a second source of allergy information used widely in epidemiological studies (19). Therefore, allergic disease outcome information was collected by two methods. (i) Doctor-diagnosed allergic events were recorded prospectively at routine or interim clinic visits and were either the presenting complaints or noted incidentally in children presenting for other reasons. The primary outcome was eczema, the most common allergic condition in infancy (17), defined as a recurrent itchy rash, with dry/scaly or wet/weeping skin, and with typical infant distribution. Doctors received support in practical dermatological diagnosis from the national referral hospital. (ii) Reported allergic events were secondary outcomes, documented during the 1-yr clinic visit by interviewing the mother or guardian using the International Study of Asthma and Allergies in Childhood (ISAAC) questionnaire (19). Data on eczema, wheeze and urticaria were collected. The ISAAC questionnaire was modified for infants by replacing ‘speech’ with ‘feeding’ and ‘exercise’ with ‘playing’. We excluded allergic rhinitis because of the difficulty in correctly distinguishing it from common colds and included urticaria for which we could obtain a clear history in the local language.

### Skin prick testing and allergen-specific IgE assays

A systematic sample of infants attending clinic visits at age 1 yr was selected to undergo skin prick testing (SPT) between September 2006 and April 2007. Subsequently, stored sera from the same infants, and obtained at the same visit, were examined for allergen-specific IgE. This data was used to assess whether infantile eczema diagnosed in this study was likely to be atopic.

Skin prick testing was carried out using standard procedures (20), with four allergens important in this setting (6) or in childhood: dust mites (Dermatophagoides mix (*D. farina*, *D. pteronyssinus*); *Blomia tropicalis*), eggs and cow's milk.

To measure allergen-specific Immunoglobulin (Ig)E, 384-well high binding microplates (Greiner bio-one Ltd, Stonehouse, UK) were coated with 15 μl/well 2 μg/ml dustmite mixture (*D. farina*, *D. pteronyssinus*) or cockroach (*Blattella germanica*) (Greer Labs, Lenoir, NC, USA) in 20 mm sodium bicarbonate/27 mm sodium carbonate buffer pH 9.6. Plates were washed four times with phosphate-buffered saline (PBS) + 0.03% Tween 20 (Sigma-Aldrich Company Ltd, Dorset, UK) and blocked with 1% w/v dried milk powder in PBS + 0.05% Tween 20. Serum samples (1/20 in PBS + 0.05% Tween 20 + 10% heat inactivated bovine calf serum) and standards were tested in duplicate. Threefold serial dilutions of specific IgE positive sera, standardised using the Immunocap assay system (Phadia, Uppsala, Sweden), formed 14 point standard curves. To six-time washed plates, 0.5 μg/ml of biotinylated monoclonal mouse anti-human IgE (clone G7-26; BD Pharmingen, Oxford, UK) was added and incubated overnight at 4°C, followed by 1/3000 streptavidin-conjugated polyclonal antibody-Horseradish Peroxidise (Mast Group Ltd, Bootle, UK), and finally 0.1 mg/ml o-phenylenediamine (Sigma-Aldrich Co. Ltd) in 0.05 m phosphate-citrate buffer, pH 5.0 containing 0.03% hydrogen peroxide. After the addition of 2 m sulphuric acid, plates were read at 490 and 630 nm dual wave length, and results interpolated from standard curves using a five parameter curve fit using Gen5 data collection and analysis software (BioTek Instruments Inc, Vermont, Winooski, USA).

### Parasitology

Stool samples were examined using charcoal culture for *Strongyloides stercoralis* and Kato-Katz method (18). Two Kato-Katz smears for each sample were read within 30 min of preparation for hookworm and after 12 h for other parasite ova. Blood was examined for *Mansonella perstans* using a modified Knott's method (18). Participants were defined as positive for a given infection if ova or parasites were seen.

### Statistical methods

Power calculations were made using disease and loss to follow-up rates from our preliminary study (17). With recruitment of 2500 women for the main trial, we expected 1860 person-years (pyrs) follow-up among infants. We expected an incidence of eczema of 25 per 100 pyr in the placebo group for either albendazole or praziquantel. This would give 80% power to detect an increase in eczema at p < 0.05 with rate ratio of 1.28 for either intervention.

The trial analysis was by intention to treat. Data were analysed using STATA version 10 (Stata Corp., College Station, TX, USA). For doctor-diagnosed outcomes, time at risk began at birth and was censored at death, loss to follow-up or at 364 days. The effect of treatment on incidence of allergic conditions was assessed using Cox regression analysis, with robust standard errors to adjust for clustering within children. Logistic regression was used to assess the effect of anthelminthic treatment on reported outcomes. Wald tests were used to assess for interactions between treatments. IgE data (transformed to log(IgE + 1) to correct skewness) and SPT positivity were each related to clinical allergy incidence by Cox regression, with robust standard errors.

Rates or prevalence of allergic conditions were first compared between treatment groups for the whole study population. Pre-specified subgroup analyses were conducted as effects were expected to be stronger when mothers had susceptible infections: albendazole versus placebo among women with hookworm; praziquantel versus placebo among women with *Schistosoma mansoni*. Differences between subgroups were examined by fitting interaction terms and applying Wald tests. All p-values are two sided with no adjustment for multiple comparisons.

## Results

**Figure 1:**
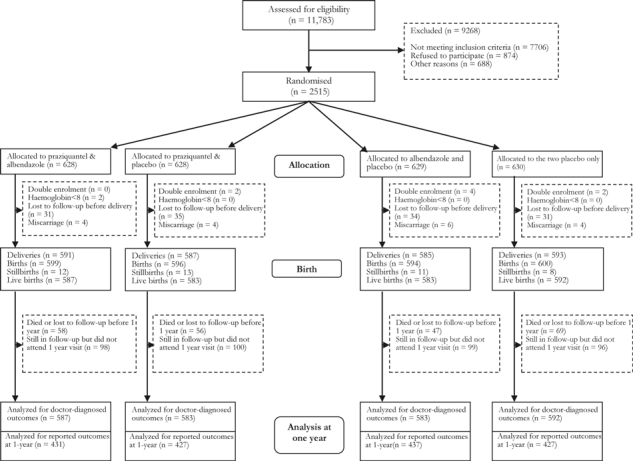
Study profile.

Recruitment and follow-up figures are presented in Fig. 1: 2507 study women were enrolled and 2201 person-years of follow-up observed among their infants.

Baseline characteristics of mothers and infants were similar across the four treatment arms (Table 1). The prevalence of worms among the pregnant women has been reported (21): 44.5% had hookworm, 21.3%*M. perstans*, 18.3%*S. mansoni*, 12.3%*S. stercoralis*, 9.1%*Trichuris trichiura*, 2.3%*Ascaris lumbricoides* and 0.5% other worms. Only 31.7% had no detected worm infection. Examination of stool samples obtained at delivery showed that hookworm and *A. lumbricoides* were effectively treated by albendazole (efficacy assuming no re-infection between treatment and delivery of 88% and 82%, respectively); *S. mansoni* was effectively treated by praziquantel (efficacy assuming no re-infection 78%); neither treatment showed significant effects on prevalence of *M. perstans, S.stercoralis* or *Trichiuris trichiura* (22). Serious adverse events (miscarriages, stillbirths, perinatal deaths, congenital anomalies) were not associated with either treatment (22).

**Table 1:** The distribution of baseline characteristics among mothers of infants included in the analysis, across the four treatment arms

Baseline maternal demographic and clinical characteristics		Praziquantel + albendazoleN = 587 n (%)	Praziquantel only N = 583 n (%)	Albendazole only N = 583 n (%)	Placebo only N = 592 n (%)
Age (years)	≤24	338 (58)	371 (64)	369 (63)	382 (64)
	>25	249 (42)	212 (36)	214 (37)	210 (36)
Education	None/primary	318 (54)	316 (54)	316 (54)	328 (55)
	Secondary/Tertiary	268 (46)	266 (46)	266 (46)	263 (45)
Household economic status	Most poor	259 (45)	272 (48)	253 (44)	269 (46)
	Least poor	318 (55)	298 (52)	321 (56)	311 (54)
HIV status	Positive	71 (12)	56 (10)	63 (11)	88 (15)
Worm status	At least one species	398 (69)	388 (68)	388 (67)	399 (68)
Hookworm status	Positive	256 (44)	272 (47)	241 (42)	256 (43)
*Schistosoma mansoni* status	Positive	107 (18)	97 (17)	115 (20)	102 (17)

### Doctor-diagnosed outcomes

Doctor-diagnosed infantile eczema was the primary outcome and most common allergic condition with an incidence of 10.4/100 person-years of follow-up (pyrs). Rates of other potentially allergy-related conditions were as follows: recurrent wheeze 0.95/100 pyrs, urticaria 1.5/100 pyrs, allergic conjunctivitis 1.3/100 pyrs, contact dermatitis 1.0/100 pyrs and papular urticaria 5.6/100 pyrs. Altogether 10,252 illnesses were recorded during infancy; total numbers of clinic visits were similar in the four treatment arms.

We found no interaction between albendazole and praziquantel treatments; therefore, we analysed the two drugs independently; that is, we analysed the effects of albendazole versus albendazole placebo in the whole study population irrespective of praziquantel treatment and *vice versa*.

Treatment of pregnant women with albendazole (compared with placebo) was strongly associated with an increased risk of infantile eczema in their offspring (Table 2). In the subgroup analysis stratified according to maternal hookworm status, the effect of treatment with albendazole on infantile eczema was slightly stronger among infants whose mothers had no hookworm than among infants whose mothers had hookworm, although this difference was not statistically significant (p-value for interaction = 0.52). In additional exploratory analyses, the effect of albendazole was also slightly stronger among infants whose mothers had no albendazole-susceptible geohelminth (hookworm *T. trichiura, S. stercoralis* and *A. lumbricoides*) [HR (95%CI) 1.99 (1.22–3.24)] than among infants whose mothers had any of these infections [1.66 (0.94–2.93)] (p-value for interaction = 0.64). Moreover, including all geohelminths, *M. perstans* and *S. mansoni*, there was a significant trend of effect with total number of worm species in the mother: the strongest effect of albendazole was among infants whose mothers had no worm infections at all [HR (95% CI) 2.21 (1.23–3.96)], compared with one species [1.87 (1.04–3.35)], two species [1.42 (0.56–3.64)] and three or more worm species [0.97 (0.19–4.86)]; test for trend p-value = 0.002.

**Table 2:** Effects of treatment with albendazole and praziquantel during pregnancy on the incidence of eczema in infancy

	Treatment	No. of eczema events	Person-years of follow-up (X100)	Incidence Rate (per 100 pyrs)	Cox HR (95% CI)	p- Value	p-Value for interaction
Albendazole treatment							
Overall effect	Placebo	83	10.92	7.33	1	0.002	
	Albendazole	148	11.09	13.35	1.82 (1.26–2.64)		
Maternal Hookworm status							
No (n = 1311)	Placebo	51	6.01	8.49	1		0.52
	Albendazole	103	6.32	16.31	1.92 (1.20–3.08)		
Yes (n = 1024)	Placebo	29	4.88	5.94	1		
	Albendazole	42	4.73	8.89	1.49 (0.81–2.74)		
Praziquantel treatment							
Overall effect	Placebo	111	10.97	10.11	1	0.80	
	Praziquantel	117	11.03	10.60	1.05 (0.73–1.50)		
Maternal *Schistosoma mansoni* status							
No (n = 1914)	Placebo	97	8.86	10.95	1		0.02
	Praziquantel	90	9.07	9.92	0.90 (0.60–1.35)		
Yes (n = 421)	Placebo	11	2.08	5.28	1		
	Praziquantel	27	1.92	14.06	2.65 (1.16–6.08)		

Treatment of pregnant women with praziquantel (compared with placebo) showed no overall effect on the risk of infantile eczema (Table 2). Among infants whose mothers had schistosomiasis at enrolment, praziquantel was associated with an increased risk of eczema, but praziquantel had no effect among infants whose mothers were not infected (p-value for interaction = 0.02).

Eighty-five per cent of hookworm infections and 65% of *S. mansoni* infections were light (<1000 and <100 eggs/gram of stool, respectively) (21). There was no statistical evidence of an increasing effect of either albendazole or praziquantel on rates of eczema with increasing infection intensity but this analysis was limited by the small numbers of participants with moderate or heavy infection intensity.

### Reported outcomes

Among 1722 infants seen at the 1-yr visit, the prevalence of each allergic condition for those with complete reported data was eczema 12% (206/1715), recurrent wheeze 9% (155/1716) and urticaria 5% (88/1708).

Maternal treatment with albendazole (compared with placebo) was weakly associated with reported eczema [OR (95% CI), p-value: 1.29 (0.96–1.72), 0.09] and strongly associated with reported recurrent wheeze [1.58 (1.13–2.22), 0.008], but showed no effect on reported urticaria [0.99 (0.64–1.52), 0.96]. When stratified according to maternal hookworm status at enrolment, albendazole showed no effect on reported eczema if mothers had hookworm [OR 1.08 (0.69–1.68)] but a slight increase if mothers had no hookworm [1.46 (0.98–2.17), p-value for interaction = 0.10]. Reported recurrent wheeze showed a similar trend, with albendazole showing no effect when a mother had hookworm [1.11 (0.64–1.91)] but a significant effect if a mother had no hookworm [1.97 (1.27–3.05), p-value for interaction = 0.31]. Praziquantel showed no effect on these outcomes.

### Skin Prick Test and allergen-specific IgE results

Out of 270 infants who had SPT at the 1-yr visit, 15 (6%) were positive to any of the four allergens (Dermatophagoides mix, *Blomia tropicalis*, egg and cow's milk). Dermatophagoides mix and German cockroach IgE assays were performed for 248 of these infants.

A positive SPT response to any allergen was strongly associated with incidence of doctor-diagnosed eczema [HR (95% CI), p-value: 6.07 (1.67–22.08), 0.006]. There were weak positive associations for both dust mite- and cockroach-specific IgE with doctor-diagnosed eczema [HR 3.28 (0.63–17.15), p = 0.16 and HR 1.79 (0.28–11.55), p = 0.54, respectively].

## Discussion

We have shown for the first time that treatment of pregnant women with albendazole is associated with increased risk of infantile eczema and that treatment with praziquantel is associated with increased risk of eczema among infants of mothers with *S. mansoni* infection. Our findings are consistent with our preliminary study (17) and support the hypothesis that maternal worms during pregnancy, or neonatal life and early breastfeeding, may protect against allergy in infancy and that treatment of these worms during pregnancy increases the risk of allergy. The effects of treatment on reported allergy outcomes at 1 yr were similar to those for doctor-diagnosed outcomes, suggesting good internal validity of the study. These results may be generalised to communities with similar prevalence and intensity of worm infections.

A possible cause of imprecision was the clinical diagnosis of eczema. Study clinicians were not dermatologists and tended to classify skin conditions that were not obviously eczema as non-specific rash. Although such misclassifications would have been balanced between treatment arms, this may have led to underestimation of the incidence of eczema and therefore to reduced power. The power of the study was lower than expected for subgroup analyses because the rate of eczema was lower than predicted, although the person-years of follow-up were greater. Both SPT and allergen-specific IgE responses of a sample of infants were positively associated with doctor-diagnosed eczema, our main outcome: these associations do not identify the causative allergen for eczema in this study, but do indicate that it was atopic in nature, and part of an atopic phenotype in these children.

Hookworm was the commonest worm species among the pregnant women and was highly susceptible to albendazole. However, the observed strong effect of albendazole among infants whose mothers had no hookworm detected was unexpected. Several possible hypotheses may be considered. First, could the effect of albendazole have been mediated through effects on undetected hookworm, or the presence of other albendazole-susceptible geohelminths in the ‘hookworm-negative’ group? The Kato-Katz method is of low sensitivity when a single stool sample is used (23), and this might be particularly important given that the majority of hookworm infections in this setting were light (24). However, among mothers in the albendazole-placebo group, 647 had at least three stool samples examined before they were treated 6 wk post-delivery; evaluation of results from all three stool samples increased the estimate of hookworm prevalence by only 6% (from 45% to 51%). We also found an effect of albendazole among infants whose mothers had neither hookworm nor other susceptible, or partially susceptible, worm infections (such as *T. trichiura, S. stercoralis* and *A. lumbricoides*). Thus, an effect of albendazole on undiagnosed hookworm infection, or other geohelminths, may not explain our result. It seems more likely that albendazole itself when used during pregnancy, or its effect on some other unidentified organism, may have had immunological effects on the foetus. This is plausible: albendazole acts by binding tubulin and inhibiting the formation of microtubules in the cytoskeleton and can affect not only worms, but also fungi, protozoa and mammalian cells (25). The observed significant trend of reduced effect of albendazole on the risk of infantile eczema with increasing number of worm species that a mother had also suggests a possible protective effect of worms against detrimental effects of albendazole. These hypotheses require further investigation. Thus, we have shown a clear adverse effect of maternal treatment with albendazole on the incidence of infantile eczema, but the role of elimination of worms in the mechanism of this effect is not certain. Further trials using anthelminthic drugs that differ in their mode of action would be useful. By contrast, for praziquantel, the detrimental effect of treatment was found only among infants of mothers with schistosomiasis, suggesting a mechanism mediated by an effect on maternal schistosome infections. This is consistent with observational results suggesting a suppressive effect of *S. haematobium* on skin prick test reactivity in children (7). Of note, in the praziquantel placebo group, the rate of eczema among infants of mothers with *S. mansoni* was lower than the rate among those of mothers without schistosomiasis (Table 2). Thus, our results indicate a protective effect of exposure to maternal schistosomiasis *in utero* against allergic disease in infancy, reversed by maternal treatment.

Our results complement observational studies (26) and animal models (27) that suggest a protective effect of maternal exposure to farm environment and to farm-related bacteria, to now suggest a protective effect of maternal worms against allergy in the offspring. Both human studies and animal models suggest that exposure to farm bacteria acts by influencing the maternal cytokine milieu and thereby modulating Toll-like receptor (TLR) expression in the placenta and foetus (28). Worm infections also influence systemic TLR expression and cytokine production (29) and might plausibly act through a related pathway.

Our randomised intervention was provided during the second or third trimester of pregnancy; all women were de-wormed approximately 6 wk after delivery. Thus, the comparison groups differed in their treatment status during pregnancy and in their worm status during the remainder of the pregnancy and the neonatal period. Differences in exposure to drugs could have elicited effects during pregnancy, and differences in exposure to maternal worms could have elicited effects either before, or immediately after delivery. However, our observations clearly demonstrate the importance of exposures very early in life for the development of the infant immune system and for the protection against allergy. Foetal or neonatal imprinting may be more important than exposures in later childhood, given the limited effect observed following anthelminthic treatment in childhood on prevalence of atopy (9). Pregnancy may provide a window of opportunity during which interventions to prevent allergy could be initiated for children at risk. Research to identify the underlying mechanisms of these effects is urgently needed.

The long-term implications of our findings remain to be determined through ongoing follow-up of the cohort and replication in other studies. A review published in 2007 reported that the risk of developing asthma in childhood for children with atopic eczema was one out of three (30). Wheeze during the first year of life may be caused by a variety of conditions and is not diagnostic of asthma, although it is of interest that anthelminthic treatment was also associated with an increase in reported wheeze in infancy in this study. The implications of our findings for the long-term risk of asthma are of greatest concern because this condition may be fatal, particularly in regions with poor health service resources (2).

Allergy prevalence is increasing globally, with no proven cause, and our results suggest that worms may play a protective role. We therefore recommend that further studies should be undertaken in areas of higher worm prevalence and intensity to investigate whether the effects we have observed in this study are replicated, and the underlying immunological mechanisms of the effects.

We have presented results of the first randomised-controlled trial on the effects of anthelminthic treatment during pregnancy on the risk of allergic disease among the offspring. We have shown that interventions during pregnancy can influence risk of infantile eczema, although the mechanism of action is not entirely clear. The detrimental effects of treatment suggest that worm infections during pregnancy or neonatal life may have a role in protecting against allergy in infancy. Worms or worm products could have a role in the primary prevention of allergic disease.
